# Exposure of feral swine to *Coxiella burnetii* overlaps with human Q fever incidence in California

**DOI:** 10.3389/fepid.2025.1692664

**Published:** 2026-01-05

**Authors:** Ian A. McMillan, Samuel J. Golon, Michael H. Norris, Gregory A. Franckowiak, James M. Grinolds, Richard A. Bowen, Vienna R. Brown, Bradley R. Borlee

**Affiliations:** 1 Pathogen Analysis and Translational Health Group, School of Life Sciences, University of Hawai'i at Mānoa, Honolulu, Hawai'i, HI, United States; 2 Department of Microbiology, Immunology and Pathology, Colorado State University, Fort Collins, CO, United States; 3 US Department of Agriculture, Animal and Plant Health Inspection Service, Wildlife Services, National Feral Swine Damage Management Program, Fort Collins, CO, United States; 4 Department of Biomedical Sciences, Colorado State University, Fort Collins, CO, United States

**Keywords:** feral swine, *Coxiella burnetii*, Q fever, seroprevalence, California

## Abstract

*Coxiella burnetii* is a zoonotic pathogen that causes Q fever in humans. There are many known reservoirs of *C. burnetii*, including cattle, sheep, and goats with an expanding list of potential reservoirs including birds, reptiles, ticks and additional mammalian species, such as swine. Feral swine are a highly invasive species in the United States with significant populations and a broad geographic distribution. The role of feral swine in the transmission and spread of *C. burnetii* is poorly understood, although a recent report identified overlap between feral swine seroprevalence and human Q fever incidence in Texas. California accounts for a large proportion of human Q fever cases in the United States and in this study we characterized the seroprevalence of *C. burnetii* in feral swine populations in the state. Feral swine showed seropositivity rates up to 1.64% indicating some level of exposure and the possibility that they may serve as a reservoir for disease transmission and spread. Overlap with human Q fever incidence was identified in the central region of California. Although this study does not directly link feral swine to human infection, it identified spatial overlap between feral swine seroprevalence and human Q fever incidence in the state of California, possibly due to the presence of ruminants as the principal reservoirs of *C. burnetii*. The environmental stability and low infectious dose of *C. burnetii*, coupled with the geographic overlap between feral swine seroprevalence and human Q fever incidence suggests that feral swine may contribute to zoonotic disease transmission and spread.

## Introduction

Feral swine are an invasive species in North America. In the early 1500s, explorers introduced swine as a source of food, subsequently leading to the establishment of feral swine populations within the modern day United States (1). Eurasian and/or Russian wild boar were introduced for sport hunting in the 1900s and domesticated pigs have also contributed to current feral swine populations in the United States. Current populations of feral swine in the United States represent a combination of Eurasian and Russian wild boar, escaped domesticated pigs, and their hybrids (2). Feral swine display a broad variety of phenotypic traits including variations in coat color, bristle length, length of nasal passages, and zygomatic breadth, yet are the same species as domesticated pigs, *Sus scrofa* (1). Common traits of *S. scrofa*, particularly in feral swine, help them survive in many environments leading to rapid population expansion. A flexible and opportunistic omnivorous diet including grasses, legumes, herbs, seeds, and other plant material increases the geographic range in which feral swine can survive (1). Populations of feral swine can increase quickly due to rapid reproduction rates and high fecundity with each mature female able to produce up to ten offspring each year (3). Decreases in predatory species in focal regions in the United States, combined with dietary flexibility and rapid reproduction rates, has led to significant geographic spread of feral swine in contemporary times (1, 2).

Feral swine are a known source of economic loss due to damage of crops and livestock throughout the world. In the United States, feral swine have contributed to major economic losses in crop producing states like Texas and California (4, 5). Predation of livestock or damage to livestock infrastructure by feral swine is another source of economic loss in the United States and other parts of the world (1, 6, 7). Overall, it has been estimated that damage and control efforts related to feral swine in the United States cost between $0.8 and $1.5 billion annually (8, 9). These estimates are likely conservative as it is difficult to characterize the economic impacts of many aspects of feral swine damage, including the costs associated with transmission of disease from feral swine to livestock, wildlife, or humans. However, a major impact that invasive feral swine have is the transmission of zoonotic diseases, like brucellosis, trichinellosis, tuberculosis, leptospirosis, and classical swine fever, to humans and/or livestock (10). Feral swine in the state of Hawaiʻi have recently been shown to have serological indications of past or current infections with *Brucella* species indicating that they may contribute to transmission of brucellosis to livestock and potentially humans (11). Increases in seroprevalence towards *Trichinella* species and *Toxoplasma gondii*, have been observed in feral swine populations throughout the United States with positivity rates rising to 12.4% and 40.8%, respectively (12, 13). Antibodies against pathogenic *Leptospira* species have been identified in feral swine of the United States with positivity rates up to 33.8%, suggesting a role in transmission of leptospirosis (14, 15). In Texas, feral swine have shown high seroprevalence rates against *Bacillus anthracis* indicating potential exposure to the causative agent of anthrax disease (16). Recently, feral swine in the state of Texas and Hawaiʻi were shown to have low but detectable levels of anti-*Coxiella burnetii* antibodies, indicating potential exposure to the pathogen that causes human Q fever (17).

Q fever is a significant disease in humans caused by *C. burnetii* that can result in severe complications including chronic hepatitis, osteoarticular infections, endocarditis, or pseudo-tumors (18–21). *Coxiella burnetii* is an obligate intracellular coccobacillus that grows in a biphasic pattern alternating between a large cell replicating form and an environmentally stable small cell, non-replicating form (22–25). *Coxiella burnetii* infects mononuclear phagocytes, replicating in a phagolysosome-like structure, and trophoblast cells of the placenta causing preterm birth and fetal mortality in animals and humans (26–30). Infected tissues and fluids from animals contain high concentrations of *C. burnetii* and have caused outbreaks of Q fever in humans (29, 31–37). Birth products, urine, feces, and milk have been shown to harbor *C. burnetii* in an increasing list of animal reservoirs including marine mammals, domestic mammals, birds, reptiles, and ticks, potentially leading to disease transmission to animals and humans (18, 28, 38–46). The infectious dose of *C. burnetii* has been reported to be a single bacterium, increasing the chances of transmission and spread from contaminated sources (47–49). Q fever is an important zoonosis with epidemic potential due to its environmental stability, low infectious dose, and numerous known/potential animal reservoirs.

Whether swine contribute to transmission or spread of *C. burnetii* to humans is currently unknown, although it is clear from serosurveys that they are susceptible to infection. Domesticated swine in South Korea, China, and Italy have shown low levels of seropositivity toward *C. burnetii* (50–52). Feral swine have also shown low levels of seropositivity to *C. burnetii* in broad geographic regions including Portugal, Spain, South Korea, and the United States (17, 53–56). In the United States, it was previously reported that feral swine in the state of California were positive for antibodies against *C. burnetii* via microagglutination test, with county level rates ranging from 24% to 68% (56). More recently, seropositive feral swine were identified via indirect ELISA in the states of Hawaiʻi and Texas, with positivity rates up to 0.19% and 6.03%, respectively (17). Hawaiʻi and Texas have contrasting rates of human Q fever that was also reflected in the seroprevalence rates of feral swine identified in each state. Overlap between human Q fever incidence and feral swine seropositivity was identified in Texas, indicating that feral swine may contribute to transmission and spread, or have a similar source of exposure to *C. burnetii* as humans (i.e., domestic ruminants) (17). Like Texas, California has a large population of feral swine and accounts for a significant proportion of human Q fever cases in the United States, accounting for ∼18% of all cases in 2019 (57). In the current investigation, we sought to survey contemporary feral swine populations in the state of California to determine seroprevalence rates and identify any overlap with human Q fever incidence that may exist.

## Results

### Characteristics of feral swine samples from California

Feral swine serum samples tested in this study were collected during routine population control efforts and archived by Wildlife Services, part of the United States Department of Agriculture, Animal and Plant Health Inspection Service. All sera from California collected between October 2019 and July 2024 were tested in this study for the presence of antibodies specific to *C. burnetii*. In total, there were 1,297 serum samples from feral swine collected from 27 counties in the state. Sample sizes ranged from one to 206 feral swine per county. Calaveras, Colusa, Sacramento, San Joaquin, Shasta, Tehama, Trinity, Tulare, and Yuba were the least sampled counties with under 10 samples/county. The most heavily sampled counties were Napa (120; 9.3%), San Luis Obispo (206; 15.9%), Santa Barbara (176; 13.6%), and Santa Clara (113; 8.7%). Swine serum samples were classified into three different groups based on age including adult (996; 76.8%), sub-adult (298; 23.0%), and unknown (3; 0.23%). There were 685 (52.8%) female pigs, 605 (46.7%) male pigs, and 7 (0.54%) pigs with no reported sex.

### Seroprevalence of *C. burnetii* in feral swine

**Figure 1:**
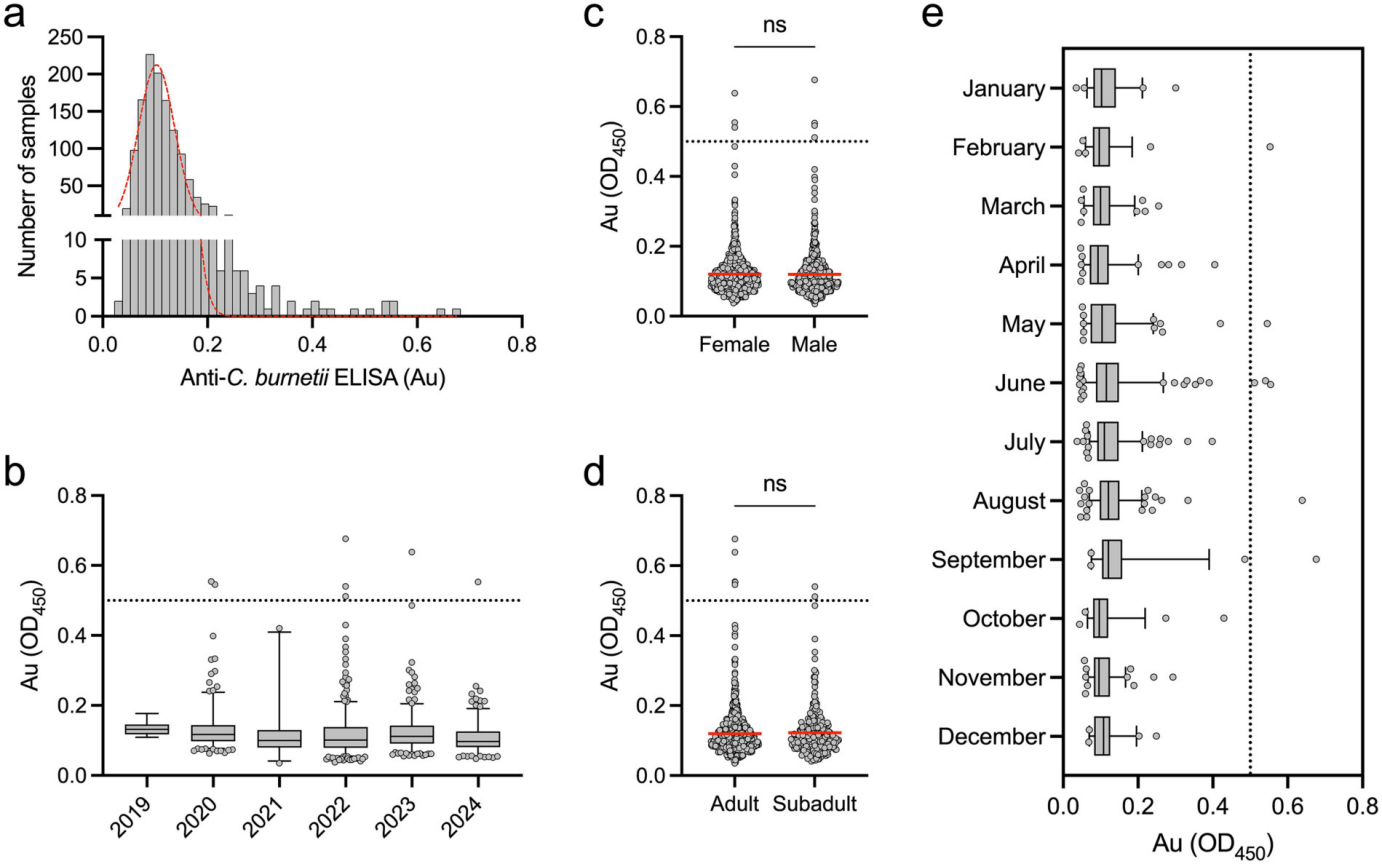
Feral swine in California show low but detectable levels of anti-*C. burnetii* antibodies. **(a)** Histogram of 1,297 feral swine sera samples from the state of California tested by indirect ELISA targeting *C. burnetii* antigens. All samples were normalized to a positive control and any absorbance (Au) value above 0.5 is considered positive. The red line shows frequency distribution of all samples following a non-normal distribution determined by Shapiro–Wilk test for normality. **(b)** Distribution of Au values for samples separated by collection year showing positive samples identified in 2020, 2022, 2023, and 2024. No significant (ns) difference was identified between Au values based on gender **(c)** or age class **(d)** determined by both Mann–Whitney U test and Fisher's exact test. e) Distribution of Au values for samples separated by collection month showing positive samples identified in February, May, June, August, and September. The positivity cutoff on graphs is represented by a dotted line at Au = 0.5. Box plots represent the interquartile range (IQR) and whiskers represent the 5th and 95th percentiles. Dots in box plots are individual samples that fall outside of the whiskers.

Feral swine have been reported to exist in 56 of 58 counties in the state according to the California Department of Fish and Wildlife. California also accounts for a large number (∼18%) of reported Q fever cases in the United States (57). Feral swine may serve as biosentinels to evaluate their potential contribution to the zoonotic spread of *C. burnetii* for this disease in the state. To test if feral swine in California have been exposed, serum samples were tested for antibodies specific to *C. burnetii*. Swine samples showed absorbance (Au) values ranging from 0.035 to 0.676 (*μ* = 0.120, *σ* = 0.061) with a non-normal distribution determined via the Shapiro–Wilk test for normality (*P* < 0.0001, Figure 1a). In the 1,297 samples tested, seven (7/1,297; 0.54%) were positive with an Au value above 0.5 indicating the presence of antibodies against *C. burnetii* (Figure 1a). The positive samples were identified in feral swine sampled in 2020, 2022, 2023, and 2024 (Figure 1b). Four of the positive samples were from male pigs and three from female pigs (Figure 1c). No significant differences were identified when Au values of gender were compared using the Mann–Whitney U test (*P* = 0.3619). In addition, when positivity and sex were compared using Fisher's exact test, no significant difference was identified (*P* = 0.7122). Five positive samples were from adult pigs and two positive samples were from sub-adult pigs and no significant difference between these two groups was identified when comparing Au values via the Mann–Whitney U test (*P* = 0.9260, Figure 1d). Positivity and age class showed no significant difference when compared using Fisher's exact test (*P* = 0.6644). Positive samples were identified in the months of February, May, June, August, and September (Figure 1e). These results indicate that feral swine have a low but detectable rate of seropositivity in the state of California.

**Figure 2:**
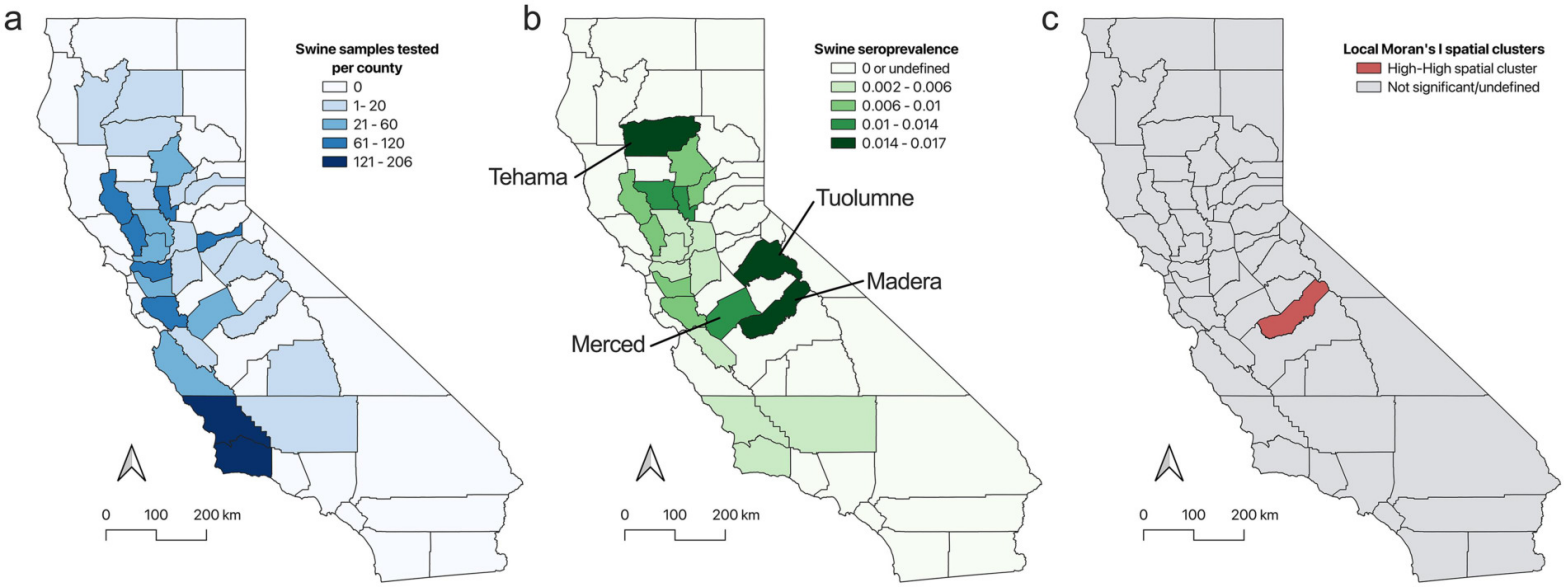
Spatial distribution pattern of *C. burnetii* seroprevalence in California. **(a)** Sample distribution across the state of California. **(b)** Spatial empirical Bayesian smoothed (SBS) seroprevalence rates for feral swine in the state of California identified the highest rates in north central Tehama County and central Tuolumne and Madera counties. **(c)** Spatial clusters characterized by LISA analysis identified a high-high cluster centered around Madera County in the central region of California.

Feral swine serum samples were collected from 27 counties in the state of California with a mean sample size of 46.25 (*σ* = 54.19) during the study period (Figure 2a). Positive samples were identified in Alameda County (Au = 0.64), Butte County (Au = 0.54), Lake County (Au = 0.51), Madera County (Au = 0.55), Santa Barbara County (Au = 0.55), Santa Clara County (Au = 0.68), and Yolo County (Au = 0.55). To better understand how positive samples were distributed throughout the sampled counties in state of California, we analyzed the seroprevalence rate for each county. Raw seropositivity rates ranged from 0% to 5.88% (*μ* = 0.56, *σ* = 1.30). Spatial empirical Bayesian smoothing (SBS) was employed to account for bias in sample sizes across counties. Smoothed seropositivity rates ranged from 0% to 1.64% (*μ* = 0.58, *σ* = 0.50). Spatial analysis identified regions with increased seropositivity rates in the central California counties of Madera, Merced, and Tuolumne (Figure 2B). SBS seropositivity rates in these counties ranged from 1.04% to 1.51% (Figure 2b). The highest SBS seropositivity rate was identified in the northern county of Tehama at 1.64% (Figure 2b). We analyzed the smoothed seroprevalence data to identify any local indicators of spatial association (LISA) using the Local Moran's I statistic. Through this analysis a significant high-high cluster centralized around Madera County was identified (Figure 2c). LISA analysis of *C. burnetii* seropositivity in feral swine populations of California show areas of increased prevalence.

### Incidence of human Q fever in California, 2014–2022

**Figure 3:**
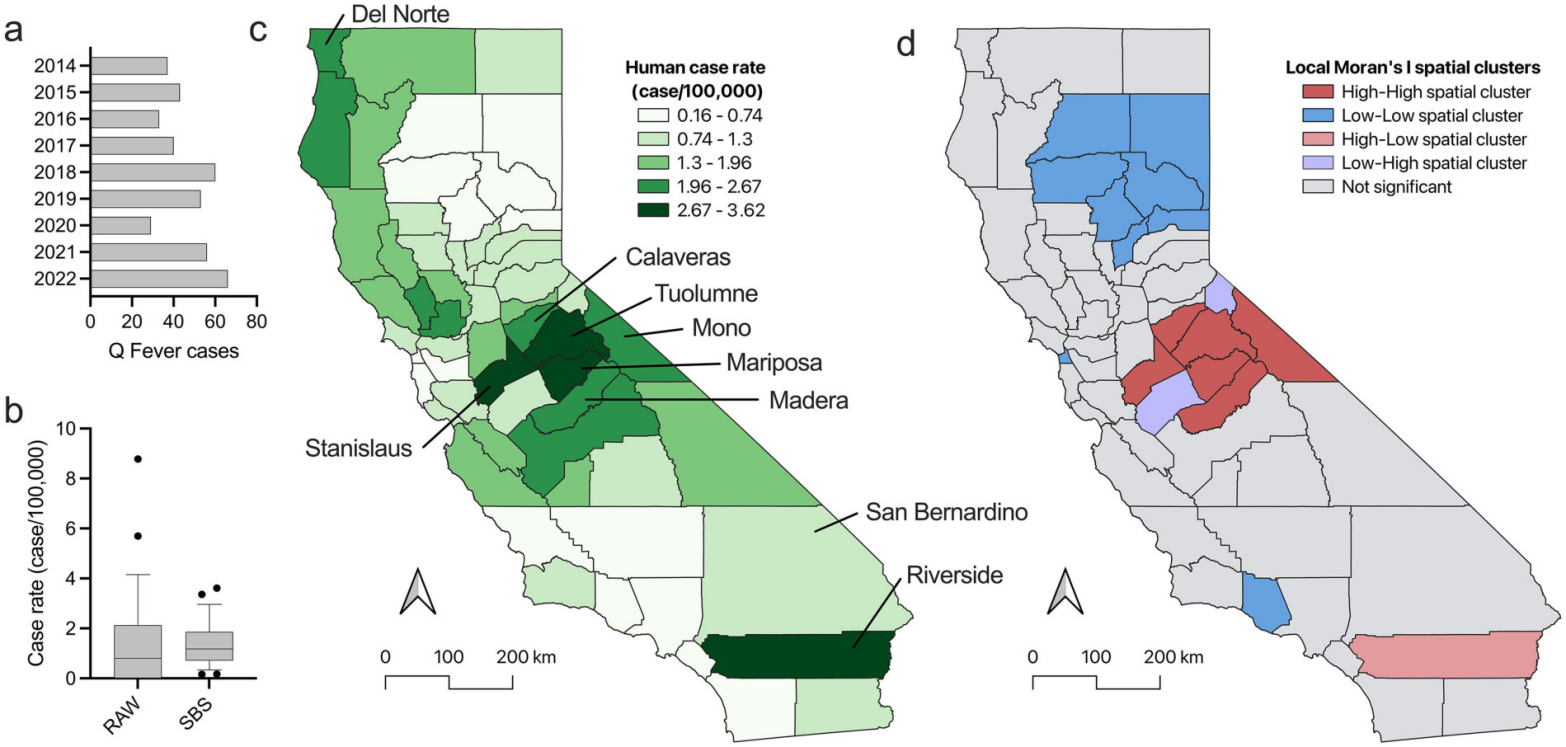
Human Q fever cases in California, 2014–2022. **(a)** Human Q fever case counts reported by the California Department of Health from 2014 to 2022. **(b)** Box plot showing the RAW and SBS smoothed case rates of human Q fever per 100,000 population in each county in California from 2014 to 2022. Box plots show the IQR with whiskers representing the 5th and 95th percentiles and dots are individual samples that fall outside of those ranges. **(c)** SBS case rates of human Q fever across the state of California from 2014 to 2022. **(d)** Spatial clusters characterized by LISA analysis identified a high-high cluster centered around Calaveras, Madera, Mariposa, Mono, Stanislaus, and Tuolumne counties in the central region of California. Low-high spatial outliers were identified in Merced and Alpine counties in central California. A high-low spatial outlier was identified in the southern region of the state around Riverside County. A low-low cluster was identified in the north eastern region of the state around Butte, Lassen, Plumas, Shasta, Sierra, Tehama, and Yuba counties. San Francisco and Ventura counties along with their spatial neighbors on the western coast were also identified as low-low spatial clusters by LISA analysis.

Q fever is a reportable disease in the state of California and any suspected cases must be reported to the local health department within one working day of diagnosis. We used publicly available Q fever count data published by California Department of Public Health to calculate rates. Between 2014 and 2022, there were 417 cases of Q fever reported in California accounting for a large proportion of all cases in the United States. Each year between 29 and 66 cases of Q fever were reported across the state (Figure 3a). Q fever was reported in 72.4% of counties with raw incidence rates as high as 8.78 cases per 100,000 population in Mariposa County. The mean raw incidence rate during this time frame was 1.35 cases per 100,000 with a standard deviation of 1.65 (Figure 3b). To account for population variance in each county, we employed SBS and identified rates as high as 3.62 cases per 100,000, with a mean rate across all counties of 1.36 with a standard deviation of 0.83 (Figure 3b). During this time period, Stanislaus County had the highest case rate in the state with 3.62 cases per 100,000 (Figure 3c). In this same region in central California, Mariposa, Tuolumne, Mono, Madera, and Calaveras counties had case rates of 3.36, 2.94, 2.67, 2.64, and 2.54 cases per 100,000, respectively (Figure 3b). Riverside County in the southern region of the state had a case rate of 2.89 cases per 100,000 and Del Norte County in the north had a case rate of 2.58 cases per 100,000 (Figure 3c). We analyzed the smoothed case rates for any clustering using local spatial association analysis via Local Moran's I. A high-high cluster was identified in central California around Calaveras, Madera, Mariposa, Mono, Stanislaus, and Tuolumne counties with two low-high spatial outliers identified in Merced and Alpine counties (Figure 3d). LISA analysis identified Riverside County as a high-low spatial outlier in the southern region of California (Figure 3d). A low-low cluster was identified around Butte, Lassen, Plumas, Shasta, Sierra, Tehama, and Yuba counties in the northeastern region of the state (Figure 3d). San Francisco and Ventura counties along the western coast were also identified as low-low spatial clusters (Figure 3d).

## Discussion

California has large populations of feral swine and accounts for the largest proportion of human Q fever cases in the United States. Texas has similar characteristics to California in that there are large populations of feral swine and a high number of human Q fever cases. The goal of the current investigation was to determine if there was geographic overlap between feral swine seropositivity and human Q fever in California. In addition, previous reports of *C. burnetii* seroprevalence in California feral swine populations showed county level rates ten times those observed in Texas (17, 56). This work, conducted over 40 years ago, used microagglutination assays to characterize current or previous exposure to *C. burnetii*, which may account for the large discrepency (56). While this technique has lower sensitivity and specificity than the techniques employed in the current study, it did identify that feral swine may be a potential reservoir for *C. burnetii* transmission and spread, a question that is still not completely understood to this day.

We found that a small percentage of feral swine in California showed serological signs of current or previous infection with *C. burnetii*. Male and female feral swine, including adult and subadult age classes, were found to be positive. Positive samples were identified in the months of February, May, June, August, and September with 71.4% of the positive samples occurring between May and August. This trend shows similarity to national observations of human Q fever temporal dynamics and previous reports of feral swine seropositivity (17, 58). While the overall positivity rate for feral swine in California was low, at 0.54%, smoothed county level rates of seropositivity as high as 1.64% were identified, consistent with reports of seroprevalence in domestic and feral swine ranging from 0.8% to 14.6% (17, 50–52). The geographically central counties of Madera and Tuolumne showed high rates of seropositivity in feral swine with the highest reported rate identified in Tehama County in the north central region of the state. A high-high cluster was identified around Madera County in central California, indicating significantly higher rates of seropositivity in this region when compared to the mean of the state-wide rate. This analysis identified regions of higher seropositivity in feral swine populations across the state of California indicating potential areas where they may contribute to transmission and geographic spread of *C. burnetii*.

While the majority of Q fever cases in humans are asymptomatic, Q fever remains a significant disease that is nationally reportable (22). Between 2014 and 2022 there were 417 cases of human Q fever reported in California with annualized raw incidence rates ranging from 0.07 to 0.17 cases per 100,000. Geographic dissection of human Q fever cases at the county level identified raw rates are as high as 8.78 cases per 100,000 between 2014 and 2022. Smoothing on a spatial scale was used to account for variations in population between counties, identifying smoothed rates as high as 3.62 cases per 100,000 over the nine-year period. The highest smoothed human Q fever incidence rates were identified in the central region of the state including Tuolumne, Mariposa, and Stanislaus counties, with 2.94, 3.36, and 3.62 cases per 100,000, respectively. Analysis of this central region also identified a high-high incidence cluster around Calaveras, Madera, Mariposa, Mono, Stanislaus, and Tuolumne counties and their neighbors, indicating that human rates of Q fever in this region were significantly higher than the overall mean for the state. Merced and Alpine counties neighbor this high-high cluster, but represent low-high spatial outliers due to low rates of human Q fever. In the southern region of the state Riverside County was identified as a high-low spatial outlier with smoothed case rates of 2.89 cases per 100,000. Other reports have highlighted numerous cases of human Q fever from Riverside and the neighboring county of San Bernadino over the last several decades (59, 60). Investigation of Q fever cases identified at the Loma Linda Veterans Affairs hospital in San Bernadino County showed that 70% of all cases reported contact with animals, and 15% reported contact with swine (60). This highlights the importance of understanding the role of animal reservoirs in the spread and transmission of this disease.

While this work does not directly link feral swine to human infection, it identified spatial overlap between feral swine seroprevalence and human Q fever incidence in the state of California. Madera County in the central region of the state showed a high rate of feral swine seropositivity towards *C. burnetii* and a high rate of human Q fever. In Madera County and its spatial neighbors, feral swine and humans are likely exposed to *C. burnetii* via the same source: contaminated birth products, urine, feces, or milk from other infected animals. All of these counties contain domesticated goats, sheep, and cattle that are known and accepted reservoirs of *C. burnetii* (18, 61). This overlap between the presence of known reservoirs, feral swine seropositivity, and human Q fever infections suggests that *C. burnetii* circulating among domestic ruminants is a likely source of exposure. Additional research is required to confirm the hypothesis that there is a shared source of infection between feral swine and humans. Other regions in the state showed a contradictory pattern. In the north central region of the state high feral swine seroprevalence rates were identified in the county of Tehama. This county and its spatial neighbors have significantly lower rates of human Q fever, the low-low cluster identified around Butte, Lassen, Plumas, Shasta, Sierra, Tehama, and Yuba counties. A similar observation was made in Texas, where feral swine in Jefferson County on the eastern coast showed high rates of feral swine *C. burnetii* seropositivity but low rates of human Q fever (17). Future work will be focused on identifying the ecological mechanisms that drive this observation where feral swine have high seropositivity and there are low rates of human Q fever.

This work identifies trends of the zoonotic pathogen *C. burnetii* relating to coincidence in feral swine and humans from 2019 to 2024 but is not without limitations. We do not directly link feral swine to human Q fever incidence and therefore can make no definitive conclusions on the role of feral swine in transmission. Feral swine were sampled from 27 out of 56 (46.6%) counties in California reported to have populations of feral swine (62). We did not survey feral swine from the southern counties of Riverside and San Bernadino, where there are high rates of human Q fever associated with animal exposures (59, 60). Targeted expansion of feral swine serosurveillance for *C. burnetii* in this region could help expand our understanding of the ecologic mechanisms supporting maintenance of Q fever in animals and its transmission to humans. We used a strict cutoff for positivity that could lead to an underestimate of seroprevalence because this approach reduces false positives. Using a lower cutoff value as other groups have done for serosurveillance would have increased the overall raw positivity rate from 0.54% to 1.54% (63). The feral swine that did not meet our strict cutoff for a positive could have waning anti-*C. burnetii* antibody titers based on their time of exposure relative to when the samples were collected. Cross-reactions to species like *Bartonella, Chlamydia, Rickettsiae,* and *Legionella* are a possibility that could lead to a false positive, however, off-target antibody titers are typically low, making misdiagnosis less likely (40). The commercial kit used in the current study has been extensively used to test swine and other animals for antibodies against *C. burnetii*, lending confidence to the results (50–52, 55, 64–66).

At present, we cannot exclude the possibility that feral swine contribute to the direct transmission of *C. burnetii* to humans, but do not provide evidence to suggest that is occurring. Here, we show that feral swine in the state of California are able to generate an immune response against *C. burnetii* infection and therefore could serve as a biosentinel, or support geographic spread and transmission to other animal reservoirs. This is consistent with other reports identifying feral swine with serological indications of *C. burnetii* infection in the states of Hawaiʻi and Texas (17). The immune responses mounted by feral swine could be due to predation of domesticated animals, as has been observed in Australia, or the opportunistic feeding of aborted birth products from infected cattle, goats, or sheep (1, 67). Contact of feral swine with contaminated urine, feces, or milk could also be a contributor to seroprevalence rates. Disease spread over time is likely facilitated by multiple factors associated with the pathogen itself, including the low infectious dose of *C. burnetii*, environmental stability of the small cell variant, and the environmental conditions that support its growth. These factors are additionally augmented by the complex interactions between wild and domesticated animals. Increased geographic range and rising populations of feral swine could potentially exacerbate risks associated with Q fever, highlighting the need for continuing population surveillance and a better understanding of the contribution feral swine have on the spread and transmission of *C. burnetii*.

## Methods and materials

### Sample acquisition

Feral swine sera was collected through a program of the United States Department of Agriculture, Animal and Plant Health Inspection Service (USDA-APHIS). The goal of the National Feral Swine Damage Management Program (NFSP) of USDA-APHIS is to help protect agricultural and natural resources, public and private property, and human health and safety by management of invasive feral swine populations in the United States. The NFSP routinely removes feral swine from environments in efforts towards this goal. During routine removal efforts, field personnel collect serum samples with associated metadata allowing for retrospective national disease surveillance and risk analysis (11, 16, 17). Serum samples studied in the present manuscript were obtained through a partnership with the NFSP. Samples were collected between October 2019 and July 2024 in the state of California (*n* = 1,297). Serum was extracted within 12 h of clotting from postmortem blood collections as previously described (11, 17). The age of male and female feral swine were classified into two groups, adult (≥1 year of age) or subadult (<1 year of age). Serum samples were shipped and stored at −80 °C at the National Wildlife Research Center in Fort Collins, CO, USA until testing.

### Serological testing and analysis

An indirect ELISA was used to determine the presence of antibodies against *C. burnetii* as previously described (17, 50–52, 55). Briefly, all samples were tested using ID Screen® Q Fever Indirect Multi-species ELISA kit (Innovative Diagnostics, France) following manufacturer's instructions in duplicate. This indirect ELISA tests for antibodies against phase I and phase II *C. burnetii* antigens and has been used to test swine serum previously (17, 50–52, 55). The specificity for this commercial ELISA kit in swine is 100% (CI_95%_: 95.58%–100%, *n* = 83) as determined by the manufacturer. The positive cutoff published by the manufacturer is 50% of the positive control and is the cutoff used in this study. Briefly, each sample's raw absorbance value at 450 nm (Au) was normalized to a positive control on the same plate (Au = 1.0) and any sample with an Au above 0.5 was considered positive, aligning with the manufacturer's cutoff. Of the 1,297 samples tested, 44.5% were tested in an additional independent experiment to ensure accuracy of the results. Normalized Au values were visually represented and analyzed in GraphPad Prism version 9.5.1 (San Diego, California, USA). Statistical analysis of Au values were carried out as described in the text using Prism.

### Human Q fever incidence in California from 2014 to 2022

The state of California publishes county level counts of human Q fever cases in yearly summaries of selected communicable diseases. This information is freely available on the California Department of Public Health webpage in the surveillance and statistics section of the Infectious Diseases Branch (https://www.cdph.ca.gov/). County level case counts were extracted from the yearly summary for 2014–2022 to analyze the spatial distribution of human Q fever in the state of California. Populations for each county were extracted from the WorldPop database (https://hub.worldpop.org/) using constrained data at a 100 m resolution for the United States in 2020 (68, 69). Case rates (raw and SBS) for human Q fever were calculated using GeoDa version 1.22 as previously described (11, 17).

### Mapping seroprevalence and incidence rates

Feral swine seroprevalence and human Q fever incidence rates were mapped to the county level in the state of California. County level GIS data was accessed from the California State Geoportal (https://gis.data.ca.gov/) and downloaded on May 9, 2024. All maps were generated using the California County Boundaries shapefile in QGIS version 3.34.3-Prizen. Raw feral swine seroprevalence rates were calculated using the number of positive samples and the number of total samples from each county. Human Q fever raw incidence rates were calculated using the number of cases and the population in each county. To account for variations in sample size or population in each county, spatial empirical Bayesian smoothing (SBS) was carried out in GeoDa version 1.22 as previously described (11, 17).

### Spatial cluster analysis of seroprevalence and Q fever incidence

Local indicators of spatial association (LISA) analysis was carried out using the local Moran's I method as previously described (70). This method identifies any high-high clusters (hot spots), low-low clusters (cold spots), or high-low/low-high clusters (spatial outliers) using a given data set. Briefly, SBS seroprevalence rates or human Q fever incidence rates were used to identify significant spatial clusters using the local Moran's I statistic in GeoDa version 1.22 as previously empolyed (11, 17, 63). The local Moran's I statistic is designed to reject the null hypotheses of spatial randomness and we used 99,999 computational permutations to determine a pseudo-*P*-value. We used a statistical cutoff value of *P* < 0.05 to determine significant spatial clusters based on a first order queen contiguity weight matrix.

## Data Availability

The original contributions presented in the study are included in the article/Supplementary Material, further inquiries can be directed to the corresponding authors.
